# Preliminary findings on the experiences of care for women who suffered early pregnancy losses during the COVID-19 pandemic: a qualitative study

**DOI:** 10.1186/s12884-024-06721-7

**Published:** 2024-08-09

**Authors:** Sergio A. Silverio, Rhiannon George-Carey, Maria Memtsa, Flora E. Kent-Nye, Laura A. Magee, Kayleigh S. Sheen, Karen Burgess, Munira Oza, Claire Storey, Jane Sandall, The PUDDLES UK Collaboration, Amy Sampson, Leonie Haddad, Elana Payne, Laura Sambrook, Venetia Goodhart, Abigail Easter, Peter von Dadelszen, Davor Jurković

**Affiliations:** 1 Department of Women & Children’s Health, School of Life Course & Population Sciences, Faculty of Life Sciences & Medicine, King’s College London, London, UK; 2 Gynaecology Services, Royal London Hospital, Barts Health NHS Trust, London, UK; 3 Gynaecology Service, Royal Free Hospital, Royal Free London NHS Foundation Trust, London, UK; 4 Department of Social Sciences, College of Health, Science & Society, University of the West of England Bristol, Bristol, UK; 5 Petals: The Baby Loss Counselling Charity, Cambridge, UK; 6 The Ectopic Pregnancy Trust, London, UK; 7 Patient and Public Involvement and Engagement Group for Perinatal Bereavement, Trauma, & Loss, King’s College London, London, UK; 8 Gynaecology Diagnostic and Treatment Unit, Elizabeth Garrett Anderson Wing, University College London Hospitals NHS Foundation Trust, London, UK; 9 Elizabeth Garrett Anderson Institute for Women’s Health, Faculty of Population Health Sciences, School of Life and Medical Sciences, University College London, London, United Kingdom

**Keywords:** Early pregnancy, Pregnancy loss, Miscarriage, Ectopic pregnancy, Molar pregnancy, Pregnancy of unknown location, Termination of pregnancy, Abortion, Qualitative research, COVID-19, SARS-CoV-2

## Abstract

**Background:**

Women who suffer an early pregnancy loss require specific clinical care, aftercare, and ongoing support. In the UK, the clinical management of early pregnancy complications, including loss is provided mainly through specialist Early Pregnancy Assessment Units. The COVID-19 pandemic fundamentally changed the way in which maternity and gynaecological care was delivered, as health systems moved to rapidly reconfigure and re-organise services, aiming to reduce the risk and spread of SARS-CoV-2 infection. PUDDLES is an international collaboration investigating the pandemic’s impact on care for people who suffered a perinatal bereavement. Presented here are initial qualitative findings undertaken with UK-based women who suffered early pregnancy losses during the pandemic, about how they navigated the healthcare system and its restrictions, and how they were supported.

**Methods:**

In-keeping with a qualitative research design, in-depth semi-structured interviews were undertaken with an opportunity sample of women (*N* = 32) who suffered any early pregnancy loss during the COVID-19 pandemic. Data were analysed using a template analysis to understand women’s access to services, care, and networks of support, during the pandemic following their pregnancy loss. The thematic template was based on findings from parents who had suffered a late-miscarriage, stillbirth, or neonatal death in the UK, during the pandemic.

**Results:**

All women had experienced reconfigured maternity and early pregnancy services. Data supported themes of: 1) COVID-19 Restrictions as Impractical & Impersonal; 2) Alone, with Only Staff to Support Them; 3) Reduction in Service Provision Leading to Perceived Devaluation in Care; and 4) Seeking Their Own Support. Results suggest access to early pregnancy loss services was reduced and pandemic-related restrictions were often impractical (i.e., restrictions added to burden of accessing or receiving care). Women often reported being isolated and, concerningly, aspects of early pregnancy loss services were reported as sub-optimal.

**Conclusions:**

These findings provide important insight for the recovery and rebuilding of health services in the post-pandemic period and help us prepare for providing a higher standard of care in the future and through any other health system shocks. Conclusions made can inform future policy and planning to ensure best possible support for women who experience early pregnancy loss.

**Supplementary Information:**

The online version contains supplementary material available at 10.1186/s12884-024-06721-7.

## Background

### Pregnancy loss and perinatal death

Pregnancy – when desired, expected, and planned – is most often associated with optimistic sentiment, leading to a positive lifecourse trajectory. A pregnancy loss, however, can act as a ‘lifecourse rupture’ and detrimentally impact the imagined future of pregnancy and parenthood as this negative trajectory is taken [1]. Every year, many millions of women, their partners, and their wider family units are affected by pregnancy loss; with worldwide estimates recording approximately 23million miscarriages and 2.6million stillbirths annually [2, 3]. In the UK, approximately 700,000 births occur each year [4]. Recent incidence rates remain relatively consistent for pregnancy loss in the UK and are as follows: 1 in 4 for miscarriage [5]; 1 in 10 for pregnancies of unknown location [6]; 1 in 54 for early elective abortions and terminations due to foetal abnormality [7];1 in 100 for ectopic pregnancies [8]; 1 in 300 for stillbirth [4]; 1 in 590 for trophoblastic disease (‘molar pregnancies’; [9]); and 1 in 660 for neonatal death [4].

The incidence rates for early pregnancy loss in the UK are likely to be under-reported as many pregnancies end in undetected loss and are, therefore, unrecorded [10], unless there is need for medical or surgical intervention and care [8]. Whilst late (second trimester) miscarriages, stillbirths, and neonatal deaths are associated with greater recording accuracy, the recording of early pregnancy loss is also thwarted by the fact that care pathways fall between primary care services, maternity care, and gynaecological and/or specialist or private early pregnancy units [11], and many early pregnancy losses occur outside the healthcare system. In the UK, Early Pregnancy Assessment Units [EPAUs] are the main healthcare service responsible for the clinical care and management of early pregnancy loss [12]; however, they vary in their organisation [13], but face significant organisational and human resource deficits which have the potential to impede the provision of high-quality, effective, and compassionate perinatal bereavement care [11].

Whilst physical health is often the primary concern of those caring for women experiencing pregnancy loss, the wider implications of the emotional burden experienced and how psychological wellbeing may be affected by pregnancy loss has increasingly been spotlighted. Pregnancy loss poses significant physical and emotional challenges [2, 14–18], and changes in clinical practice have seen mental health included within clinical care pathways [19]. The associated grief and trauma of pregnancy loss has been reported as lasting an indeterminate amount of time, as often being latent in nature, and can reprise at future lifecourse transitions, such as during a subsequent pregnancy [20]. Psycho-social support is a known protective factor against the longer lasting mental health impacts of perinatal bereavement [21, 22], as are the networks of partners, family and friends, and healthcare professions (including specialist counselling, psychological, and mental health support as appropriate) who bereaved mothers may draw upon [17].

### The global COVID-19 pandemic

The SARS-CoV-2 pandemic shocked health systems globally and forced overnight reconfiguration of services to reduce the spread of infection. In the UK, maternity and gynaecological care including early pregnancy care and subsequent perinatal bereavement care services underwent substantial reorganisation [22–26]. In practice, this meant a drastic reduction in the time and resources dedicated to maternity care provision [23, 24, 27–30], perinatal mental health services [31, 32], and perinatal bereavement care [22]. Patients testing positive with COVID-19 and requiring medical intervention took priority in hospitals, and staff were redeployed to manage the burgeoning ‘COVID-19 wards’ [27]. Many perinatal care consultations were conducted virtually [24, 31–33], partners and birthing companions were often prohibited [34–36], and universal face-mask policies were in place [22, 24]. During this period clinical staff working in maternity care and gynaecological settings were generally overworked and care was highly protocolised (i.e. following prescribed and sanctioned ways of working based on the principle of infection control), potentially limiting empathy [22, 24, 27, 28]. Much written about perinatal experiences during the pandemic concedes poorer experiences from pregnancy to postpartum [34, 37–41]; a notable decline in perinatal mental health care [31, 32]; and sub-optimal care for women and their partners after a perinatal bereavement [22]. To date, however, there has been no assessment of how women have perceived and experienced the changes made to the NHS provision of early pregnancy loss care during the pandemic.

### The present study

This study is part of The PUDDLES *(‘Experiences of Parents who Suffer Pregnancy Loss and Whose Babies Die during the Pandemic’*) Study programme of work – a global collaboration aiming to understand the experiences of those who suffered a perinatal bereavement during the pandemic. In the following analysis, we present data about experiences of service reconfiguration and care provision from women in the UK who suffered one or more early pregnancy losses during the SARS-CoV-2 pandemic. This analysis contributes the first evidence from the UK on how reconfigured early pregnancy loss services were experienced by women.

## Methods

### Study design

The PUDDLES Study programme of work is strictly qualitative, adopting all the parameters of in-depth qualitative work and therefore is philosophically situated within a qualitative research paradigm. We adopted an ontologically critical realist [42] and objectivist epistemological [43] perspective. This means reality of the lived experiences is argued as being in observable existence albeit beneath the participant narrative, and therefore may not always be recounted as an exactly accurate representation of the event itself. This is often due to participants being (un)consciously influenced by social constructs, norms, and expectations, which may in turn affect one’s behaviour or recollection of the event to appear more socially desirable or in an attempt to seek validation for actions or emotions from the researcher [44]. Nevertheless, this narrative becomes and is the participant’s lived reality and so is accepted as their truth.

### Patient and public involvement and engagement

This work was formed as a sub-study of The PUDDLES Study – a nested qualitative study within the wider COCOON Global Collaboration [45], which brought together collaboration from several countries to investigate the impact of COVID-19 on new, expectant, and bereaved parents and the care they received during the pandemic. PUDDLES is investigating the experiences of late-miscarriage, stillbirth, neonatal death, and associated care during the SARS-CoV-2 Pandemic. It is one of many UK-based studies which feeds directly into PIVOT-AL – a national collaborative of academics, researchers, clinicians, policy makers, and members of charitable organisations [46]. This group is dedicated to investigating the effects and outcomes of the pandemic on mothers, babies, their families, and healthcare professionals in maternal and child health settings and services which have been reconfigured during the health system shock, re-organised in para-pandemic times, and as they start post-pandemic recovery. At a meeting of PIVOT-AL in November 2021, two areas of research were identified as being unaddressed by current or ongoing pandemic-related portfolios of research: early pregnancy loss and early elective abortion care. This study was therefore devised in response to a call from PIVOT-AL for researchers to plug this lacuna.

To ensure sensitivity and appropriateness of recruitment materials and interview schedules, and to aid recruitment, The PUDDLES Study team originally worked with the International Stillbirth Alliance, Tommy’s Charity, and Sands (*‘Stillbirth and Neonatal Death Society’*). Subsequently, PUDDLES – Early Pregnancy Loss has worked in conjunction with Petals: The Baby Loss Counselling Charity, The Ectopic Pregnancy Trust, and The British Pregnancy Advisory Service. Through all these engagements, we received feedback on recruitment, study design, and interpretation on findings from both lay and expert stakeholders, including members of the public, those with lived experience, health and social care professionals, researchers, and policy makers. Further details of patient and public involvement and engagement within this programme of work can be found at the end of the article.

### Recruitment and participants

Women were recruited to the study via on-line social media posts shared by researchers and collaborating charities (Petals: The Baby Loss Counselling Charity and The Ectopic Pregnancy Trust) in March 2022. Recruitment adverts were removed from our charitable partners’ social media platforms within three days of posting, due to the overwhelming response of more than 60 expressions of interest. All interested persons were invited to interview and were screened for eligibility, criteria for which included: women, aged ≥ 18 years, who had experienced at least one early pregnancy loss during the SARS-CoV-2 pandemic (i.e., since 30 January 2020 and until data collection ceased in June 2022). All eligible participants who expressed interest and responded to scheduling contact were interviewed, with the remainder who did not maintain contact to organise an interview date, assumed to have decided to not participate. By offering interviews to all who expressed interest, we removed any researcher-based selection bias from our recruitment to the study. Full demographic details of the participants can be found in Table 1*,* and details of the pregnancy losses can be found in Table 2*.*

**Table 1:** Participant Demographics

**Demographics**		**N/32**	**Percentage of Dataset**	**Demographics**		**N/32**	**Percentage of Dataset**
**Age (Years)**	<18	0	0.0	**Parity**	Nulliparous	19	59.4
	≥18 but <20	0	0.0		Primiparous	10	31.3
	20-24	0	0.0		Multiparous^b^	3	9.4
	25-29	6	18.8	**Gravidity**	Primigravida	15	46.9
	30-34	8	25.0		Multigravida	17	53.1
	35-39	11	34.4	** Planned Pregnancy**^**c**^	Yes	23	71.9
	40-44	5	15.6		No	9	28.1
	45-49	2	6.3	**Had COVID-19**^**d**^	Yes	21	65.6
	≥50	0	0.0		No	11	34.4
**Geographical Location**^a^	London, England	4	12.5	**Ethnicity**^**e**^	White (incl. English, Welsh, Scottish, Northern Irish, or British; Irish; Gypsy or Irish Traveller; Any other White background)	29	90.6
	North East, England	0	0.0		Mixed/Multiple Ethnic Groups (incl. White and Black Caribbean; White and Black African; White and Asian; Any other Mixed or multiple ethnic background)	2	6.3
	North West, England	4	12.5		Asian/Asian British (incl. Indian; Pakistani; Bangladeshi; Chinese; Any other Asian background)	1	3.1
	Yorkshire and the Humber, England	2	6.3		Black/African/Caribbean/Black British (incl. African; Caribbean; Any other Black/African/Caribbean background)	0	0.0
	East Midlands, England	1	3.1		Other Ethnic Group (incl. Arab; Any other ethnic group)	0	0.0
	West Midlands, England	2	6.3	**Religion**^**f**^	Christian (all denominations)	9	28.1
	East of England	7	21.9		Roman Catholic	4	12.5
	South East, England	5	15.6		Jewish	1	3.1
	South West, England	3	9.4		Muslim	0	0.0
	Wales	0	0.0		Hindu	1	3.1
	Scotland	2	6.3		Sikh	1	3.1
	Northern Ireland	1	3.1		Agnostic	1	3.1
	Other (incl. islands)	1	3.1		Any Other Religious Belief	0	0.0
**Marital Status**	Married	16	50.0		Atheist or No Religious Belief	15	46.9
	Co-habiting	13	40.6				
	Single (incl. Divorced, Never Married, & Widowed)	3	9.4				
	Alternative Relationships (incl. Polyamory; Platonic Communal Living)	0	0.0				

^a^Geographical location based on International Territorial Level – a geocode standard used by the Office of National Statistics in the United Kingdom

^b^All multiparous women had two living children

^c^Two participants were on waiting lists for IVF, but fell pregnant with the pregnancy they subsequently lost

^d^COVID-19 diagnosis could have been at any time, not just during pregnancies and any which were reported as ‘maybe’, but undiagnosed, were counted as ‘yes’

^e^Ethnicity was self-defined by participants and subsequently assigned to the list held by the Office of National Statistics devised for the 2021 Census

^f^Religion was self-defined. Where participant was non-practising, but gave their religious heritage, the religion was recorded

**Table 2:** Participants’ Pandemic Pregnancy Losses and Dates

Participant ID	Pregnancy Losses	Date of Pregnancy Loss	Participant ID	Pregnancy Losses	Date of Pregnancy Loss
P-EPL-001	Early Miscarriage	November 2021	**P-EPL-017**	Miscarriage with Ovarian Ectopic Pregnancy (Suspected Twin Pregnancy)	September 2021
P-EPL-002	Ectopic Pregnancy Early Miscarriage	January 2021 March 2021	**P-EPL-018**	Early Miscarriage Ectopic Pregnancy	April 2021 November 2021
P-EPL-003	Early Miscarriage Early Miscarriage Early Miscarriage	June 2020 January 2021 January 2022	**P-EPL-019**	Early Miscarriage Pregnancy of Unknown Location Chemical Pregnancy	November 2020 February 2021 July 2021
P-EPL-004	Ectopic Pregnancy	September 2020	**P-EPL-020**	Ectopic Pregnancy	January 2021
P-EPL-005	Early Miscarriage	July 2021	**P-EPL-021**	Early Miscarriage	March 2022
P-EPL-006	Early Miscarriage	March 2022	**P-EPL-022**	Chemical Pregnancy Ectopic Pregnancy Ectopic Pregnancy	December 2020 April 2021 July/August 2021
P-EPL-007	Early Miscarriage Early Miscarriage	June 2020 August 2021	**P-EPL-023**	Ectopic Pregnancy Early Miscarriage Ectopic Pregnancy	December 2020 November 2021 April 2022
P-EPL-008	Early Miscarriage Early Miscarriage	August 2020 May 2021	**P-EPL-024**	Ectopic Pregnancy	February 2021
P-EPL-009	Ectopic Pregnancy	July 2020	**P-EPL-025**	Ectopic Pregnancy Early Miscarriage Early Miscarriage Ectopic Pregnancy	August 2020 January 2021 April 2021 August 2021
P-EPL-010	Ectopic Pregnancy Chemical Pregnancy	February 2021 July 2021	**P-EPL-026**	Termination of Pregnancy Early Miscarriage	June 2021 January 2022
P-EPL-011	Early Miscarriage Termination of Pregnancy Pregnancy of Unknown Location Early Miscarriage	March 2020 September 2020 March 2021 August/September 2021	**P-EPL-027**	Ectopic Pregnancy	November 2021
P-EPL-012	Ectopic Pregnancy	November 2020	**P-EPL-028**	Molar Pregnancy	August 2020
P-EPL-013	Early Miscarriage Early Miscarriage	July 2020 January/February 2021	**P-EPL-029**	Ectopic Pregnancy	September 2020
P-EPL-014	Early Miscarriage Early Miscarriage Early Miscarriage Early Miscarriage	May 2020 June 2020 September 2020 March/April 2021	**P-EPL-030**	Ectopic Pregnancy	July 2021
P-EPL-015	Early Miscarriage Early Miscarriage	October 2020 March 2022	**P-EPL-031**	Early Miscarriage Early Miscarriage Chemical Pregnancy Molar Pregnancy	March 2020 June 2020 November 2020 April 2021
P-EPL-016	Ectopic Pregnancy	February 2022	**P-EPL-032**	Ectopic Pregnancy	February 2022

### Data collection

The interview schedule (see Additional file 1) was developed [SAS, FEK-N] in-line with earlier PUDDLES-UK work [22] and in consultation with the wider team [LAM, DJ] and charitable organisations [Petals: KB; The Ectopic Pregnancy Trust: MO]. The form and format of the interview schedule was subject to refinement, having previously been deemed acceptable for this type of study in earlier PUDDLES-UK and PUDDLES-Global work [22]. Interviews were conducted between March and June 2022 [by SAS (*n* = 6): a male expert on qualitative interview techniques with responsibility for qualitative innovation and training; and FEK-N (*n* = 23): a female Master’s student who had undergone intensive, advanced qualitative methods training and worked under supervision [from and by SAS – which helped for standardisation of interviewing technique]; or with both interviewers (*n* = 3)], via video-conferencing software. Interviews covered experiences of early pregnancy loss (including multiple losses, if appropriate), access to and engagement with services, and support networks. They were semi-structured in nature, to allow flexibility of questioning, whilst ensuring common questions were asked and therefore were comparable across the dataset [47]. Interviews were recorded (*M*_*Time*_ = 71min), and audio files were transcribed, verbatim. Analytical notations or ‘memos’ were added, in writing, to the base of each interview, post-transcription.

### Data analysis

Template analysis [48–50] is regarded as being philosophically flexible [50], but is highly methodical as a method of analysis, following a six-step process: 1) Re-familiarization with the Data; 2) Preliminary Coding; 3) Organization of Themes in the Template; 4) Defining the Template; 5) Application of the Final Template to the Full Dataset; and 6) Finalization of Template Definitions; whilst also engaging with critical reflexivity (i.e. questioning and querying any biases being introduced by the analyst – be they interpretive or in quotation selection, and where biases are thought to have occurred: noting them, bracketing them off from the analysis, and holding them in suspense, until they can be interrogated as part of the interpretation phases of the write-up) and iterative analysis (i.e. going back over previous coding and sense-checking each transcript against the global pool of selected quotations), to ensure rigour [49].

The template itself was adopted and adapted from the related, earlier, PUDDLES study which focused on late-miscarriage, stillbirth, and neonatal death [22]. This method of adaptation of an existing coding template was utilised as a method of investigation in order to be able to draw comparisons of experience between the extant group (i.e. those who suffered a later pregnancy loss or perinatal death) and the present study population (i.e. those who suffered an early pregnancy loss). By basing the thematic template on the previous findings from the same study and therefore controlling the context within which the phenomenon occurred (i.e. both during the pandemic), interpretation can be more easily drawn than if the data had been coded organically through a more inductive methodology.

**Fig. 1:**
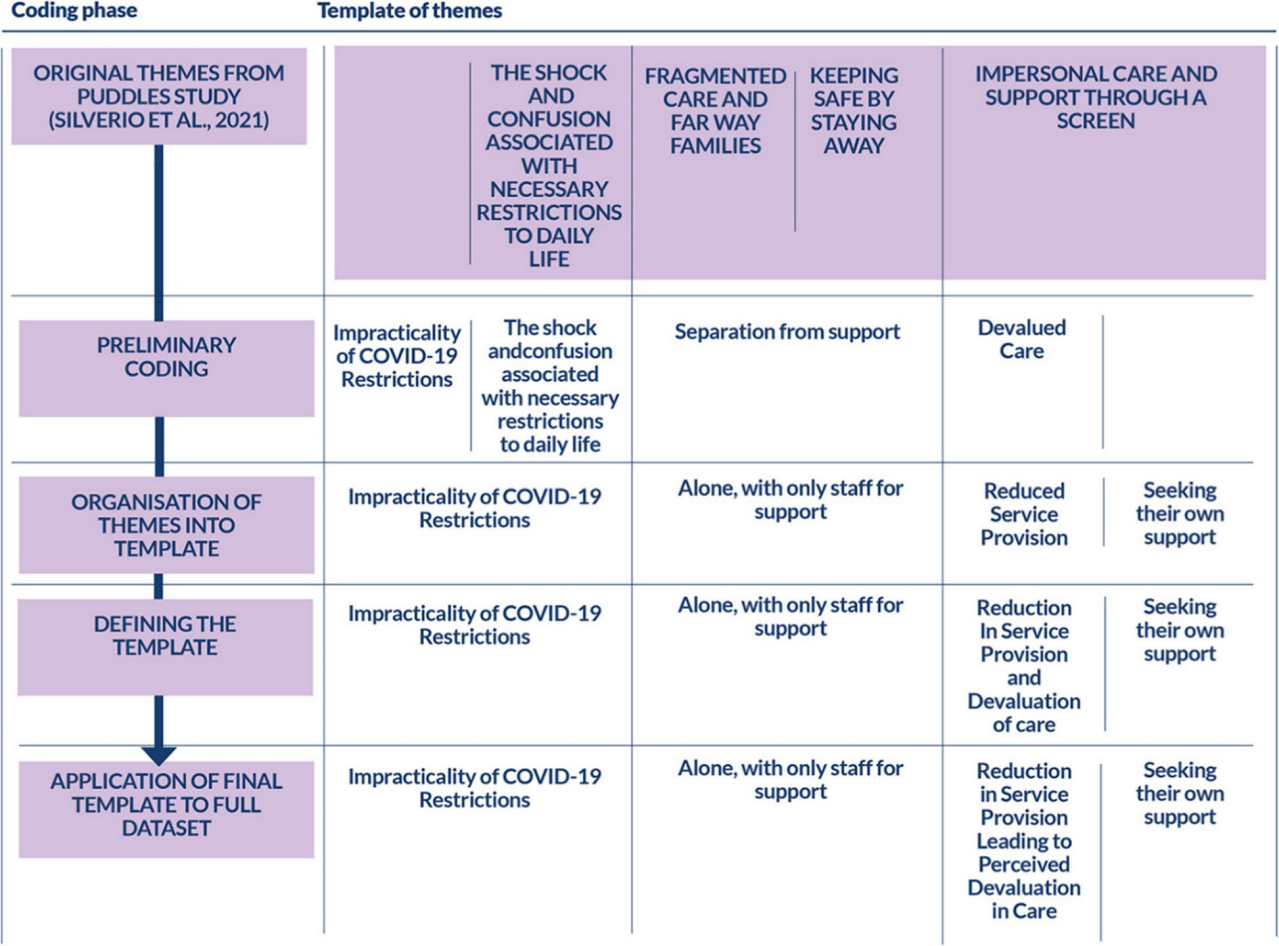
Template of Themes

Analysis began with re-reading of all the transcripts in the dataset to ensure good familiarity with the data. Coding was iterative [SAS, RG-C, MM] and conducted in NVivo – initially being grouped under one theme called ‘Service Reconfiguration due to COVID-19’ [RG-C, MM], and then re-coded at a more granular level [SAS], into the themes from the previously published PUDDLES Study [22] which were: 1) The Shock & Confusion Associated with Necessary Restrictions to Daily Life; 2) Fragmented Care & Far Away Families; 3) Keeping Safe by Staying Away; 4) Impersonal Care & Support Through a Screen. The rationale for basing the analysis on these previous findings was to allow for the comparison of experiences between later perinatal bereavements (as identified in Silverio et al., 2021 [22]) and early pregnancy losses presented in this study. Themes were augmented through iterative analysis, to better suit the early pregnancy loss context and then iteratively revised (*see *Fig. 1), resulting in 4 themes: 1) COVID-19 Restrictions as Impractical & Impersonal; 2) Alone, with Only Staff to Support Them; 3) Reduction in Service Provision Leading to Perceived Devaluation in Care; 4) Seeking Their Own Support.

Two axes of saturation were assessed: data saturation and thematic saturation. We assumed data saturation when new transcripts added to the dataset did not present any additional thematic lines of inquiry (i.e. the themes did not change in scope and no new themes were required to address content of the data), and this was achieved after 21 transcripts. We accepted completion of thematic saturation, when the themes were adequately supported by data from across the dataset an could therefore be recognised as standalone themes (i.e. there was an abundance of representative data for each final theme), and this was achieved at 11 transcripts. Iterative selection of quotations rendered only the most illustrative being utilised in the analysis below.

## Results

We present findings from the second cycle of PUDDLES-UK qualitative interview data collection (March 2022) which focused on early pregnancy loss during the pandemic (the first cycle of PUDDLES-UK data collection happening between November–December 2020 and being focused on later perinatal bereavements). These qualitative findings from the UK take account of the service reconfigurations and re-organisation of perinatal care, providing a thorough appraisal of the changes to care which happened as the pandemic continued. We aim to provide points of learning for the safe recovery and re-build of early pregnancy loss services in the aftermath of the SARS-CoV-2 pandemic and for future health crises.

### Impracticality of COVID-19 restrictions

The first theme generated in this analysis focused on how women described the restrictions of the pandemic as being at best impractical and inconvenient, but more often and more problematically impersonal and inadequate, when trying to access early pregnancy loss services:

*I can't know for sure because I haven't had this experience outside of COVID. But I know it made me feel, in a certain way. Like I said, it made me feel like I was more of a bother. It made me less inclined to seek out medical help than perhaps I would have been normally. It made everything so much more of a ball ache. Like, I've got to go and wait in A&E for four hours now for this, and do I really want to go to A&E and wait for four hours? Not really. It was just that kind of thing. And then, obviously, not being able to see people's faces at a very emotional point in your life, I would never have thought that that would be quite as bad as it was, but because they're just strangers anyway, and your eyes are where the people have the emotion, but just not being able to see mouths, I don't know what it is. It makes everything seem so much more clinical than it would have, I think.* (P-EPL-010)

The pandemic-related restrictions were also reported to have made accessing care more emotionally difficult after suffering a loss. Many women reported experiences marked by insensitivities, especially when forced to utilise areas in the hospital in which pregnant women and newborns were frequently present. This could be seen to demonstrate a ‘double hardship’ amongst women who were faced with the emotion surrounding a pregnancy loss and then felt they were left without any emotional scaffolding because of the way that services were being delivered:

*The hospital where I am, they have two lifts. With COVID, that is really restricted, what you can go in and out……… We sat at that lift waiting to get on, on and off, constantly seeing babies, constantly seeing pregnant people……… That didn’t help me……… I just went really downhill after that.* (P-EPL-015)

There was a distinct feeling amongst women that the staff were having to keep to strict protocol for care delivery – which jarred with the nature of their caring profession. Often, COVID-19-related restrictions were perceived as interfering with healthcare professionals’ ability to deliver care as they would have wanted to:

*I got the sense that people wanted to give you a hug, or just to place their hand on your arm, but they weren’t allowed to do that. So, there was always a distance between you.* (P-EPL-014)

*When I turned up the day after I was told I’d had a missed miscarriage, I was upset and they said, “Oh I’m really sorry, I can’t hug you”. So just that acknowledgement of what they would like to do but that they can’t, just the empathy…* (P-EPL-007)

The reporting of unnaturalness about the effect of the restrictions on care delivery was common, and some women explained how the strangeness of navigating the healthcare system, with its associated restrictions, at a time of health crisis made things more difficult:

*If I compare it to when I had my first pregnancy loss back in 2017 when I had to have surgery, the masks, everything felt like it was out of a sci-fi film. Yes, really strange, really surreal.* (P-EPL-027)

### Alone, with only staff for support

Whilst the pandemic was deemed impractical, it was the result of these restrictions, which often rendered women alone during their pregnancy care. One of the major changes to all maternity care services was the exclusion of partners from appointments, scans, and labour wards (until active labour was established). For women who suffered an early pregnancy loss, the situation was the same with partners being excluded most of the time:

*…went for the what was the 12-week scan. Due to COVID, I had to do that all on my own. I mean, they made it very clear that partners couldn’t come in……… I felt I couldn’t contact my partner because of all the messages, all the signs everywhere. I think because of COVID they didn’t want you to get your phone out. So that made the situation quite difficult.* (P-EPL-007)

This meant women were often reliant on healthcare professionals – in addition to providing their clinical care – to provide the physical and emotional support and reassurance in the absence of other sources of support usually provided by a partner, family member, or friend, even if this meant the healthcare professional was in breach of the protocol for care provision, infection control, and social distancing at the time:

*…with the first miscarriage, when I got taken away to the pregnancy unit, and they were giving me my options of what I should do because of my missed miscarriage, I was on my own and I had nobody to talk to about it. And I wasn’t allowed to have anybody with me. And, again, when I had the MVA [Manual Vacuum Aspiration] – which, by the way, is the most painful thing I’ve ever experienced I was holding the hand of a little nurse who I didn’t know.* (P-EPL-003)

The absence of a partner at the time of the pregnancy loss sometimes caused issues afterwards. Where partners were absent whilst women were processing and grieving their loss, they reported it often made partners more detached from the reality of it and harder for them to understand:

*I think, for me, because he wasn't there, and still now I feel like because he wasn't there going through it, we don't really talk about it. And I feel like it's kind of because he wasn't going through it. It's almost like it didn't happen for him.* (P-EPL-029)

On rare occasions, partners were allowed to be present so that women were not having to go through their medical and surgical procedures alone, but this was at the discretion of the staff and it was made known that this was in breach of the guidance:

*My husband was not allowed in the hospital. He… the nurse was actually so worried about me that she called my husband to say, ‘We have absolutely no visitors because of COVID, but as an exception, we can allow you to come in,’ and at that point we were both worried that that was because I was at risk of not making it through the night, so this was on the Sunday night. So,* <*Husband’s Name*> *came in, held my hand and we just sat and cried through full PPE on the Sunday evening.* (P-EPL-024)

Women were cognisant of the turmoil many staff seemed to encounter when they were unsure how to provide care in the way they usually would and how they had to adhere to strict infection control protocols – with the two often being interpreted as being in contravention to one another. Many women recalled it was often one member of staff in particular who was especially kind to them during their loss, which made being alone more bearable whilst under their care – even if the staff themselves were visibly awkward about how best to support women given the pandemic situation:

*I will say that the midwife who did that scan was absolutely lovely and very supportive, and I remember when she said that, that ‘I can confirm it has been a complete miscarriage’, she put her hands on mine actually. And I remember thinking oh, I’m not sure you’re allowed to do that because of COVID rules [laughs]. I mean, she was wearing gloves, obviously, et cetera. But I was kind of like, I don’t want her to get into trouble. But it was a very sweet thing to do.* (P-EPL-005)

*I think the midwife was a bit wary of how to speak, so it made the sentences take a long time to come out and there’s a lot of very sad eyes. I don’t know how to explain it. It’s just… I just need the facts and then to be asking the questions and then to move on. But I understand that a lot of other people would want someone to, I don’t know… They did some nice things, like even though they were wearing gloves, they’d touch your hand, or just touch you, which is, I think, quite nice when you’re in a situation where you’re not really seeing anyone and you’re not allowed your partner in, so it feels really isolating. They were okay, nothing horrible was said.* (P-EPL-023)

### Reduction in service provision leading to perceived devaluation in care

Women generally understood the need for restrictions, however, like being made to be alone during their care, the reduced service provision was often perceived as a reduction of high-quality (and sometimes even, basic) care. The most troubling of the findings derived from women’s experiences of care for early pregnancy loss during the pandemic therefore were the reports of the unequivocal reduction in service provision, which more often than not led to the perception that care was of poorer quality:

*Found out I was pregnant and then about ten days after I found out, it wasn’t planned, we were in a lot of shock but ten days after I found out, I was in a lot of pain, excruciating pain. So, I called 111 [non-emergency NHS number] and they weren’t very helpful at all. They put me through to a nurse who said ‘I’m not a midwife and I’m not a doctor, so I don’t know what you want me to do for you. You need to book in with your GP. If you’re having a miscarriage, you just need to ride it out’. And then just ended the call.* (P-EPL-004)

*So, my experience was that the pandemic, I think, had quite a significant impact on the care that we received. Now, I’ll obviously have to counter that by saying, I haven’t had a miscarriage not during [i.e. outside of] COVID, so I don’t how that would differ……… But certainly, I was told, when I rang the early pregnancy unit, that one of the reasons why it was taking a very long time for someone to get back to me was because they had staff off sick, so they were having staffing shortages.* (P-EPL-005)

The restrictions had wider implications, including on staffing, where healthcare professionals were taking time off due to COVID-19 infection. Overall, whilst women understood the staffing issues and the restrictions, they consequently were often recipients of care lacking in relationality or sensitivity:

*So, I did not get sympathetic or understanding care in the early pregnancy unit at all [pauses]… The whole experience was very traumatising. The complications that I just touched on around whether it was ectopic or not, et cetera, meant that I had to go back for repeated blood tests for quite some time, which was very retraumatising. At one point, I was waiting in the waiting room while the nurse was eating a bacon sandwich [laughter] and I went up to the window and said I was here for my blood test and she said, ‘Yeah, yeah, take a seat,’ and she was eating a bacon sandwich and she finished her bacon sandwich, got up, came out and said, ‘Sorry to keep you waiting. Actually, I am not sorry because I needed my breakfast because we are all run off our feet because there are no staff because of COVID,’ so that touches on the COVID issue.* (P-EPL-017)

Recognition of the impact of broader healthcare interactions on early pregnancy loss care is essential. There was quite a high reporting of women seeking scans for reassurance at private clinics because they were unable to access EPAU care, as with the participant’s quotation below. This often meant that the transfer from private settings to NHS ones were implicated in the same service delivery issues as reported within the hospitals themselves. Despite optimal clinical care within early pregnancy services, negative experiences of loss can arise from interactions with other healthcare professionals outside the EPAU:

*The staff at the clinic were amazing, the ambulance staff were horrendous, and I did put in a complaint actually, which is very unlike me, but I remember them coming in and asking me why I was crying. I was like [laughter] ‘Why do you think?’ Like ‘why are you crying?’ What? And then in the ambulance, I think I had some paracetamol, or I had already had some and he said, ‘No, this will not be an ectopic pregnancy. If it was, you would be screaming. You would be in so much more pain than this if it was ectopic. It is really sad that you are miscarrying, but… but the puzzling thing was that at the scan, they had given us printouts of the ultrasound and a letter saying, ‘We can see a mass in the left fallopian tube and fluid in the abdomen, suspected ruptured ectopic, urgent referral to A&E’ and he looked at it and went, ‘No, no, that is not what it will be because you would be in way more pain. You would probably even be unconscious by now if that is what it was.’ Within an hour, I was in surgery having my fallopian tube removed and I was like, ‘What a dick. What an absolute dick!’* (P-EPL-025)

As is common in the extant literature-base on care and safety in gynaecological and maternity, women in this study frequently reported the devaluation of care actually centring on being demeaned or dismissed – often by singular individuals amongst the healthcare staff:

*Then she said, “Because you’ve told me you are in pain, I’m going to have to scan you”. [Sighs]. Made me feel really degraded. There was no compassion in what she did.* (P-EPL-012)

*I think they needed beds, didn’t they, they wanted people out, so it was kind of ‘let’s go do the ward round, tell her that…’ Didn’t really give me any information about what I felt, and it was kind of, ‘get her out the door’.* (P-EPL-004)

Whilst all people seeking healthcare should be treated with care and compassion, women in this study did intimate that the pandemic circumstances almost gave license to some healthcare professionals to not engage in the emotional care women desired at this emotional point of both physical pain and psychological anguish. This regularly led to women feeling like they themselves were or their pregnancy loss was an inconvenience to the healthcare system:

*I would have liked to have been treated as a person for a start. That would have been nice. And not an inconvenience.* (P-EPL-017)

*That’s what I would like. Just for it to be treated like it’s something real as opposed to an inconvenience.* (P-EPL-008)

With some members of clinical staff working in strict adherence to the pandemic-restrictions imposed onto healthcare settings, women regularly reported on how staff treated those around them, including partners, family members, and even their children. This was often recalled as not being at all compassionate, and included guarding of certain clinical spaces, monitoring of time, and exclusion of chosen and consented chaperones (e.g. partners, friends, or family):

*COVID was very much a thing. The sonographer refused to let my daughter come into the room with me. And she was left outside with a stranger. Now bearing in mind, at the time she would have been two and a bit, so she’s left outside with a stranger, screaming her head off. I mean obviously I’m quite distressed because I’m bleeding and the sonographer is not telling me good things, but she just refused to let her come in. So that was all really, really distressing.* (P-EPL-011)

The lack of compassion demonstrated by some staff exacerbated women’s distress, particularly when they were trying to manage their own emotions and those of their family members at this difficult time; and women often responded performatively in an attempt to not cause additional burden to the staff who they recognised were working at more than capacity, and were often burnt-out:

*I think I would have liked the ward to be more compassionate. I would have liked the ward staff to be more empathetic to the situation. I would have liked* <*partner name*> *to be able to come in with me. They did tell me if I was there seven days he could come in for an hour, which made it feel a bit like a… like you are just on holiday, it’s like, you know, you’ve got to build up your loyalty points* (P-EPL-012)

### Seeking their own support

To combat the impracticality, loneliness, and perceived reduction in care, many women turned to their own networks of support. This often linked directly to the perception of devalued care where many women felt they were having to make decisions about their care pathways and future reproductive health in the absence of their partners who were not being allowed to be with them:

*I basically made the decision whether to have the surgery or not with two other women in the ward who were both having treatment for ectopic pregnancy. Sorry, one actually had a suspected ectopic, but her baby was alive, and another one who was in for something else, and a really very, really, really good friend phoned me in the morning and we just made the decision, which was quite a big decision to make, but I was really isolated in making it, and really the only people that I really had to talk to were kind of those that were available to chat, so that friend and the nursing staff and others on the ward.* (P-EPL-022)

Furthermore, when it came to aftercare, many women discussed how their hospital often did not provide them with any onwards signposting. Whilst this is not necessarily unique to the pandemic circumstances, it was definitely exacerbated as psychological support was no longer available on-site and in face-to-face format, therefore those seeking aftercare had to manage with on-line-only provision:

*And interestingly, no-one from the NHS signposted me to The Ectopic Pregnancy Trust, or to certain… No-one. The next day, I was waiting for the gynae team to come in, just to check I was okay so I could be discharged. They literally said nothing. They were like ‘You'll be fine in a week to go back to work, you'll be alright. You lost one tube, but you'll be fine, you'll get pregnant again.’ It was so blasé. ‘And off you go.’ No signposting, no support, no support for mental health whatsoever, so I had to do a lot of that research on my own.* (P-EPL-027)

This meant women were either expected to manage the psychological consequences of their pregnancy loss and their decisions about future reproductive health alone or had to find their own resources:

*I managed to, via Google, find Petals, the baby loss charity, who offers counselling, and that was just off my own back, and managed to get myself booked into some sessions there. I think there was about a seven to eight-week wait. But then managed to start seeing them and they’ve been brilliant and transformational and I’m actually still talking to my counsellor through Petals. And that’s been hugely positive. All of that has been virtual, because of the pandemic but that to me, that hasn’t detracted from the experience at all in fact that’s probably made it easier to access because that’s made it easier for me to work around work and childcare et cetera. So, in that sense actually the remote nature of pandemic care has been really helpful in terms of accessing the mental health support through Petals.* (P-EPL-005)

The on-line nature of support was typically discussed negatively, which led some women to seek alternative and innovative forms of support and information. Here we see that the pandemic increasingly forced the locus of responsibility of particularly psychological care, shifted to women almost without exception:

*The main podcast that I’ve listened to is the ‘The Worst Girl Gang Ever’ which sort of started because of someone having a miscarriage during COVID and feeling like there wasn’t any support……… And then the Miscarriage Association support groups were originally held in person but because of COVID they are now on-line, and they were once a month……… the first group I joined I didn’t say anything, I turned the camera off. I was literally a fly on the wall, which you couldn’t do in an in-person meeting, and I might have got more out of an in-person meeting, potentially, but I don’t think I’d have made the step to go [laughs], so the fact that it was available on-line got me there. And even if it only got me 50% of support it was 50% is better than 0% kind of thing.* (P-EPL-006)

Amid the pandemic, although women understood the necessity of psychological and social support to be on-line, many who had experienced a pregnancy loss expressed a preference for 'in person' support had it been available:

*…they do the Sands group in a 45-minute drive away in person. For me, it was actually better that it was online. I don’t know if I would have braved, in the shitty place that I was, actually going to meet a load of people that I knew probably would already know each other, for the first time. So, in a way, that made it easier for me to access that support, in the group dynamic. The counselling, I think I would have probably preferred that to be face-to-face, but obviously that wasn’t how it was, because of COVID.* (P-EPL-015)

## Discussion

The pandemic-related restrictions which were placed on public life, coupled with those which changed how healthcare was delivered in the NHS, profoundly altered how early pregnancy care was delivered. The exclusion of consented partners (i.e. the friend, family member, or romantic partner who the woman wants to accompany her and agrees to be present during consultations) from care settings has been consistently highlighted as problematic [22, 24, 41, 51]. In the context of pregnancy loss, partners can act as a source of support, someone to retain and relay information, and as an advocate [52–54]. In this study, the feeling of having to navigate the loss alone in the absence of their partner was thought to amplify women’s traumatic experiences. This was especially the case with regards to the distress experienced at the point of being informed about their loss, which echoes the experiences of mothers who were bereaved by later pregnancy losses and perinatal deaths during the pandemic [22]. Similarly, the idea of having to navigate the maternity healthcare system alone during the pandemic has been found in mothers of live infants [41, 51], suggesting the effect of the restrictions to maternity care were not only felt by those suffering a loss, and were holistically punitive [37]. This is particularly pertinent given that the levels of psychological distress for pregnancy loss can vary [55], may last an indeterminant amount of time, and is known to affect women at different stages of their post-bereavement lifecourse [2, 11, 17, 56], meaning the long periods of isolation whilst receiving care as was required during the pandemic [57], were especially damaging for women experiencing early pregnancy loss.

The availability and quality of early pregnancy loss care was of significant concern in this study. Women repeatedly discussed how both the availability of care was affected by the pandemic and it exacerbated existing issues in pre-pandemic care [11–14, 58–60]. This widespread drop in quality of services within of maternity care during the pandemic has been reported previously and has centred around reduction of provision, choice, agency, and a feeling of compromised safety due to the reconfiguration of services and reduced staffing [28, 30, 37]. It is, however, especially disappointing for early pregnancy loss care given the efforts the sector has gone to in order to improve bereavement care services with the implementation of The National Bereavement Care Pathways [19] and evidence-based recommendations which have previously highlighted the systemic shortcomings of pre-pandemic care offer [12]. Given this guidance and these recommendations were formulated just prior to the pandemic, it is possible that the health crisis we found ourselves in, hindered their implementation, thus rendering services unable to make positive change to the system, structure, or provision of care.

The combination of a reduced service provision, restrictions which demanded partners were excluded and likewise put distance between women and their attending staff, and ongoing issues with staffs’ approaches to care may have magnified the struggle to cope with the unprecedented circumstances they faced at a time of personal tragedy. It has previously been reported, partners of bereaved women have a different psychological response to the pregnancy loss [15, 61–64]. However, the physical separation of partners from all aspects of early pregnancy loss care may have further removed partners from connecting with the experience emotionally, and exacerbated the potential for women to feel isolation, failure, and lack of support during this time [14]; thus, having a profound negative impact on relationships [65, 66]. In summary, in 'normal' (vs. pandemic) circumstances of early pregnancy loss care, the act of partners being together in the healthcare setting usually fosters unity and bonding, enhancing their ability to support one another through their shared experience of the early pregnancy loss and associated grief.

The COVID-19 restrictions were definitely reported as adding another layer of distance between women and their support networks, with most women stating they had to seek their own support – especially professional support. This resonates with the findings from work with mothers who suffered a late-miscarriage, stillbirth, or neonatal death during the pandemic [22]. The predominance of an on-line offer for post-bereavement care and support was evaluated with mixed endorsement, in-line with other findings from work conducted across maternity services during the pandemic [22, 24, 32, 37, 51]. Like their counterparts who suffered a later pregnancy loss, stillbirth, or neonatal death [22], some women who suffered an early pregnancy loss found the COVID-19 restrictions to be ‘double-edged’. In one sense, the restrictions provided women with an opportunity to extricate themselves from distressing social situations and making the home a ‘safe space’ – free of potential triggers – in which they could grieve at pace and in a place they set. In another sense, restrictions which removed the social and familial networks away from the settings in which women grieved meant they were increasingly isolated and had to rely on healthcare professionals for psychological, emotional, and sometimes even physical support, in addition to the clinical care they were providing. Here, we can interpret the actions of healthcare professionals as stepping into these empty roles which could not be filled by women’s relatives and loved ones due to the pandemic restrictions [21], and so became the only and correct people to do so given the circumstances. Moving forward, thought should be given to whether these socio-emotional roles which go beyond usual, compassionate, and relational care, are going to be expected of all healthcare professionals as an extension of the holistic provision [67], or whether it is unsustainable, unrealistic, and over-reaching of healthcare professionals’ expected duties.

### Strengths, limitations, and future directions

This study benefits from capturing data from the height of the pandemic health system shock to provide pertinent knowledge about women’s experiences of care from early pregnancy loss services in the UK, during the COVID-19 global pandemic. The analysis we present provides the first insight into the impact of how the pandemic changed how early pregnancy loss services were delivered and offers recommendations for how best to navigate future health crises, whilst still delivering high-quality care. It is imperative that future, global research works to understand the impact of the pandemic and rallies efforts to agree on minimum standard of care provision in perinatal bereavement services, when faced with global and/or local health system shocks. This study is not without limitations, with one being the form of the present, preliminary analysis, which adopts a descriptive form, based on solely UK interview data, which may not be directly transferable to other health settings, particularly low- and middle-income settings or countries where healthcare is not free at the point of access. Future research should also consider collecting education status to provide a more holistic insight to participant demographics. Nonetheless, the ongoing PUDDLES Global Collaboration will aim to tackle this issue by collecting, analysing, and publishing data from other countries, as it becomes available; as well as more in-depth analyses of data from the UK. Furthermore, we have a predominantly White population in this study, which is a drawback to our findings, given that pregnancy loss effects all women from all ethnicities and women from Black, Asian, and Minority Ethnic backgrounds continue to have complex relationships with healthcare settings [68], especially in the perinatal period [69]. We also recognise White women in the UK generally find access healthcare more readily and navigate healthcare services more easily than minority ethnic women [32, 69–72] and those who do not have English as a first language [73, 74]. We posit the uptake of participation in this research amongst ethnic minority women may mirror that of uptake of psychological support after loss amongst ethnic minority women, which also remains low [70]. Future research should also dedicate efforts to specifically investigating the experiences of ethnic minority women [71, 75]; and those living with co-morbidities [76] and/or disability, who are known to experience perinatal mental health difficulties differently to women with no known disability. We would also recommend purposeful investigation into the experiences of sexual minority women who suffer a pregnancy loss and who have a different perinatal mental health profile to heterosexual women [77]. It would also be important to undertake comparative studies in diverse healthcare settings, such as private healthcare in countries which have a free-at-point-of-use system, healthcare where you must pay-for-use, and healthcare in low- and middle-income settings. Quantitative data would also be useful to understand both the epidemiology and psychometric profile of those suffering early pregnancy losses on a national and international level. A final area for future research to focus on is women who had an early elective abortion or termination of pregnancy due to foetal anomaly, as these women are also under-represented in The PUDDLES Study to date.

## Conclusion

The COVID-19 pandemic was an unprecedented and uncontrollable global event. Within it, many women went on to suffer an early pregnancy loss – as it were – one significant lifecourse rupture within another. The compounded grief of life as we once knew it, of the pregnancy, and of the imagined future for the baby women thought they were going to have, made for a situation in which women found themselves alone, having to navigate challenging pandemic restrictions and revised early pregnancy loss care pathways, within a depreciated and rapidly devaluing care system. We argue, therefore that consented partners should be deemed an essential part of early pregnancy loss care, and their presence should never face forced removal through any health system shock, unless it is at the request of the woman or when the partner poses potential risk to themselves, the woman, other patients, or healthcare staff (i.e. there is known domestic violence and/or abuse; drug and alcohol problems; etc.). Furthermore, devaluation of care by individuals due to their demeanour, tone, or approach to women in their care, remains indefensible – and the NHS must do more to improve sensitive patient management for perinatal bereavement of all forms. We must remember, however, that most healthcare professionals who were providing face-to-face care during the pandemic – particularly during early days – did so with the knowledge they may acquire a fatal infection as a result of undertaking their job.

In stark contrast to the findings of this work, research conducted in The PUDDLES Study focusing on mothers who suffered a late-miscarriage, stillbirth, or neonatal death suggested a greater degree of lenience or tolerance towards the presence of partners. This may be linked to the process of having to give birth to the baby in the case of later pregnancy losses and stillbirths, and in the case of a neonatal death, there being a period of time where the baby is alive, although often in an intensive care unit. Alternatively, the expected management of early pregnancy losses differs from that of later perinatal deaths, with women suffering early losses expected to wait in crowded waiting rooms and are admitted to open wards; whilst women suffering a stillbirth or expected neonatal death are admitted to individual rooms on the labour ward which facilitates privacy whilst being looked after by a single midwife. However, our findings suggest the quality of care provided for mothers who suffered an early pregnancy loss was more significantly depreciated than those who suffered a late-miscarriage, stillbirth, or neonatal death. We caution that healthcare services should therefore be mindful to not create a ‘first among equals’ status whereby the care for later pregnancy losses is retained at a higher level than for early pregnancy losses when there is a health system shock or other healthcare crisis.

### Supplementary Information

Additional file 1. The PUDDLES-Early Pregnancy Loss Study Interview Schedule.

## Data Availability

The datasets generated and/or analysed during this study are not publicly available due to the sensitive nature of the interviews, but a de-identified dataset may be available from the corresponding author upon reasonable request.

## Abbreviations

EPAU: Early Pregnancy Assessment Unit (a specialist unit that provides care for women with problems in early pregnancy)
MVA: Manual Vacuum Aspiration (a surgical procedure performed with local anesthetic, after an ultrasound scan suggests there is remaining pregnancy tissue inside the womb following a miscarriage)
NHS: National Health Service
PPIE: Patient and Public Involvement and Engagement
RCOG: Royal College of Obstetricians and Gynaecologists
SARS-CoV-2: Severe Acute Respiratory Syndrome Coronavirus 2 (a.k.a. COVID-19)
UK: United Kingdom
WHO: World Health Organization
